# Evaluating digital transformation in small and medium enterprises using the Alkire-Foster method

**DOI:** 10.1016/j.heliyon.2025.e41838

**Published:** 2025-01-10

**Authors:** Luís L. Moreira, Sofia S. Pinto, Leonardo Costa, Nuno Araújo

**Affiliations:** a Universidade Católica Portuguesa, Católica Porto Business School, Portugal; b Universidade Católica Portuguesa, Católica Porto Business School, Research Centre in Management and Economics, Portugal; c CATIM - Technological Centre for the Metalworking Industry, Portugal

**Keywords:** Industry 4.0, Digital transformation, SMEs, AF method

## Abstract

The digital technology transition fosters the flexibility of companies and their ability to respond quickly to market changes. However, Small and Medium-sized Enterprises (SMEs) seem to be particularly challenged by digital transformation. The Alkire-Foster (AF) method was employed to analyze the integration profiles of digital technology in SMEs. Individual and group average partial and composite indicators were computed, as well as group measures of digital technology integration, to benchmark individual companies and groups. Additionally, a linear regression was used to examine how individual overall composite scores of digital technology integration are influenced by dimensions of individual digital technology integration enablers and other contextual variables. The results indicate that most companies possess the digital infrastructure necessary for this integration. However, there is room for improvement in terms of supply and demand of digital skills. This study contributes to research by applying an existing tool, the AF method, in a novel context: measuring digital technology integration in SMEs. The AF method facilitates effective benchmarking at both individual and group levels.

## 1 Introduction

In the last decade, digital technologies, platforms, and infrastructure have been transforming the way society works in significant and disruptive ways [1]. Technological transitions change technology, regulations, infrastructure, organizations, and culture [2]. For instance, digital technologies such as autonomous robots, 3D printing, or the Internet of Things (IoT) have been associated with increased demand for skilled labor and the replacement of unskilled workers [3]. Originally labelled by the German economic development agency German Trade & Invest (GTAI) as Industry 4.0, the fourth industrial revolution results from the emergence, advancement, and convergence of several technologies that foster a real-time connection between the physical and the digital realms [4]. Such a technological transition fosters the flexibility of companies and their ability to respond more quickly to market changes.

Reference [5] evaluates the value and return on investment of digital solutions for small and medium-sized enterprises (SMEs). The findings highlight several benefits, including increased efficiency, cost reduction, productivity growth, enhanced customer satisfaction, and a competitive advantage. Despite the potentially tremendous benefits, SMEs are lagging behind in their integration of digital technology [6,7].^1^ SMEs play a crucial role in driving innovation, economic growth, and job creation [6].

Companies are confronted with changes taking place in the business environment (reshaping supply and/or value chains), business strategy, and organization [8,9,10,11,7]. Demand has become more volatile and oriented towards low-cost and customized mass production, with unforeseen challenges brought by the COVID-19 pandemic, the Russia-Ukraine conflict, and the war in Gaza and the Middle East. Supply and competition have increased with globalization and digital transformation. To overcome the challenges, companies need to be more flexible and able to respond more quickly to market changes [12], which requires new business proposals and plans [13,11]. On the one hand, start-ups and large companies are leading in the integration of digital technology, and on the other hand, SMEs seem to be particularly challenged [6]. As they have fewer internal resources, SMEs rely more on the networks they integrate into their innovation processes [14,15,16]. Given the importance of SMEs in the European economy for business and employment,^2^ it is important that these companies successfully integrate digital technology to at least stay competitive in the Digital Society economy. Despite this, research into the factors driving the adoption of digital technologies by SMEs has been limited [17,18].

This research aims to assess and benchmark the stage of digital technology integration in companies of the Portuguese metalworking industry, an industry dominated by SMEs. Are SMEs lagging behind in their integration of digital technology? Do they lack digital technology infrastructure and supply and demand of digital skills? How are these factors enabling digital technology integration? Does the type of governance of the value chain in which SMEs participate, namely in Global Value Chains (GVC), affect their digital technology integration? These are the research questions we have put forward. To address them, the Alkire-Foster (AF) method and linear regression were used.

The AF method can be applied to microdata. It is particularly suitable for evaluating and comparing SMEs and groups of SMEs in relation to digital technology integration. In this process, at the company level, the partial indicators from the European Commission's Digital Transformation Scoreboard (EC DTS) have been adapted and extended in order to develop, using the AF method, individual and group average partial and composite indicators for companies, as well as group measures.

An online questionnaire was also carried out with companies in the Portuguese metalworking industry. By generating profiles of digital technology integration, and individual and group average partial and composite indicators, as well as group measures, the AF method makes it possible to identify the most critical intervention areas, with practical implications for management and policy. Together with the regression analysis performed, it has enabled responses to the research questions to be gathered and some hypotheses identified in the literature to be tested, namely that SMEs are lagging behind in their integration of digital technology. It has also allowed testing the impact of factors enabling digital technology integration in companies.

The paper is presented as follows. Following the introductory section, section 2 presents a literature review on Industry 4.0 and the challenges it poses for SMEs, the EC DTS, which was used to develop the partial indicators, and the AF method. Section 3 outlines the methodology used, including the development of the questionnaire, the calculation of individual and group average partial and composite indicators, the creation of group measures, the application of the questionnaire, and the performance of OLS regression to support the benchmarking exercise and test factors that enable the integration of digital technology in companies. Section 4 presents and discusses the results. Section 5 concludes the paper by summarizing the main findings, detailing the contributions to academia, management, and policymakers, and outlining the limitations and providing suggestions for future research.

## 2 Literature review

### 2.1 Industry 4.0

There are many definitions of Industry 4.0. According to reference [19]p. 2925, “Industry 4.0 is operationalized as the usage of intelligent products and processes, which enables autonomous data collection and analysis, as well as interaction between products, processes, suppliers, and customers through the internet”.

Industry 4.0 involves the digital transformation of the industrial sector [20]. It began as the name of a German cooperation project involving the government, the private sector, and academia to boost the next era of manufacturing and establish Germany as a leader of integrated industry [21]. Industry 4.0 enables technology innovation in manufacturing systems, aiming to enhance productivity, efficiency, and growth [22]. Companies adopting Industry 4.0 aim to achieve three critical production targets: flexibility, quality, and productivity [23].

Industry 4.0 is also referred to as the industrial internet of things (IIoT) [9]. It has emerged from the digital transformation of industrial processes, combining Information and Communications Technology (ICT) with digital manufacturing technologies, joining the physical world of industrial production with the digital world, generating a digitalized and interconnected industrial production known as the cyber-physical systems (CPS), in which heterogeneous data are shared between the physical environment and the digital environment [24]. It comprises the adoption of disruptive digital technologies and principles, such as social media, mobile services, cloud computing, IoT, cybersecurity solutions, blockchain, robots and automated machines, big data and data analytics, 3D printing (also known as AM - additive manufacturing), artificial intelligence (AI), augmented reality, virtual reality, CPS, and smart factories [24,25,9,26,27].

Reference [28] explores how manufacturing industries can gain competitive advantages through innovation and the adoption of digital technologies. It encompasses various digital tools and techniques, presenting four main concepts as common denominators of Industry 4.0: (i) digital technologies, both hard (Robotics or AM) and soft (AI or machine learning); (ii) digitalization and data; (iii) innovation; and (iv) new business development. They assert that all four domains are interrelated and can be analyzed both individually and as part of Industry 4.0. Using this structure, this research project aims at analyzing the level of integration of digital technologies and the role of some enabling factors.

Although Industry 4.0 has been attracting attention from governments, industries, and researchers, many of its aspects are still unknown and uncertain [27]. For instance, Ref. [29] suggests that the digital transformation of SMEs is not only about integrating digital technologies, but also about rethinking their business models. Integrating digital technologies can lead to improved performance through innovative business models [29]. The present study aims to contribute to a better understanding of the level of integration of digital technologies in this context.

### 2.2 Challenges for SMEs to implement industry 4.0

Given the importance of SMEs in the European economy for business and employment, it is important that these companies successfully integrate digital technology to stay competitive.

Reference [30] reviews factors facilitating the digital transformation success of SMEs. It emphasizes the importance of understanding their starting points, including limitations and unique characteristics. Digital transformation strategies should be tailored and gradual. The study highlights continuous investment in education and learning, including training employees on new digital tools and processes to ensure they are well-equipped for the transformation. Strong leadership commitment and management support are critical, as is focusing on customer needs and preferences.

Reference [13] examines how Industry 4.0 influences business model innovations in German SMEs. The authors find that Industry 4.0 impacts Value Creation, Value Capture, and Value Offer, with the company's role and motivation influencing which elements are innovated. SMEs are categorized into four types - Craft manufacturers, Preliminary stage planners, Industry 4.0 users, and Full-scale adopters - regarding their Industry 4.0 strategies.

Reference [31] examines how different types of digital ecosystems affect the innovation and growth of SMEs. The authors conclude that SMEs can achieve high levels of innovation and growth by aligning their strategic orientation, resource base, and network position with the characteristics of these digital ecosystems.

Reference [32] shows that the adoption of Industry 4.0 technologies improves the technical performance of Korean SMEs.

Reference [22] provides a review of the interplay between technological innovation and Industry 4.0 paradigm. Some studies suggest that SMEs that achieve higher levels of digital diffusion experience an increase in their innovation performance over time [33].

Reference [34] examines the impact of digital orientation on SME performance, discovering a U-shaped relationship. Initially, increased digital orientation may lead to performance setbacks, but once a certain threshold is reached, performance improves. This indicates that SMEs must strategically manage resources and develop the necessary capabilities to navigate digital transformation successfully.

Reference [15] conducts a review and focus group studies in the USA, Italy, Austria, and Thailand, identifying six types of challenges in adopting the referred technologies:

- *Economic/financial* - The lengthy process of implementing Industry 4.0 may require extensive and costly investment. SMEs often do not have a clear perception of the market potential and/or economic benefits of Industry 4.0 and face liquidity constraints.
- *Cultural* - SME top management demonstrates a desire to safeguard their autonomy, coupled with a lack of vision, risk aversion, resistance, and lack of support vis-à-vis Industry 4.0. The country's organizational culture/mentality can lead to a lack of cooperation between company functions/departments and between companies. Resistance from employees, due to limited knowledge of Industry 4.0 and the fear of losing their job.
- *Skills/resources* - Lack of qualifications, training, and technical knowledge of managers and employees. The complexity of Industry 4.0 requires SMEs to find suitable research partners and/or to participate in open innovation (OI) environments [35]. As such, because they have fewer internal resources, SMEs depend more on their networks for innovation [16]. However, SMEs may have difficulties in joining and/or forming networks with other companies. And when participating in value chains, particularly in GVCs, they may come across chain governance that does not facilitate their adoption of Industry 4.0.^3^
- *Legal -* The main challenges concern cybersecurity. However, some restrictions need to be considered that are imposed through the regulations and laws governing countries.
- *Technical* - SMEs often lack technical standards, are technologically immature and have a fragile and/or unreliable Information Technology (IT) infrastructure. Different ICT systems, data stored in different silos that do not communicate with each other, and buildings not designed to automate internal transport are some of the technical issues that many SMEs face.
- *Implementation process* - The need for new business models and proposals, new methodological approaches, and coordination efforts. Sometimes, the absence of roadmaps and/or toolkits for the implementation of Industry 4.0.

Reference [36] finds that SMEs leverage business networks to develop three dynamic capabilities that enhance their adoption of non-disruptive digital technologies and improve their value proposition and competitiveness: human resource capabilities, strategic planning capabilities, and marketing capabilities. Among these, marketing capabilities have the most significant positive impact, while human resource capabilities negatively affect adoption.

Reference [37] examines the challenges and solutions for SMEs in emerging economies adopting digital platforms. Key barriers include existing laws, low governmental support, mismatches between business needs and platform features, and limited access to digital technologies.

Reference [38] analyzes the challenges and mechanisms of digital transformation in SMEs within developing countries. The review highlights that basic information systems are the most adopted technologies in SMEs within developing countries, while more advanced technologies like cloud computing and artificial intelligence are less prevalent.

Reference [39] reviews the digital transformation in SMEs, highlighting a shift towards agile processes. Key technologies like cloud computing, big data, and IoT enhance efficiency and decision-making. Strategies such as digital marketing, e-commerce, and CRM improve customer engagement and streamline operations. Challenges include the lack of digital transformation frameworks, limited empirical studies, and the need for more research on leadership's role, as well as financial constraints and resistance to change.

Reference [40] examines the factors influencing the adoption and evolution of digital marketing strategies in SMEs. Market pressures compel SMEs to adopt digital marketing to remain competitive, while organizational readiness dictates the effectiveness and extent of this adoption.

Reference [41] investigates the impact of digitalization on management control systems in Italian SMEs. The authors find that digitalization significantly enhances company performance and communication. Key drivers for successful digitalization include top management support, human resources, and financial resources. Medium-sized companies are more proactive in digital transformation. The research also underscores the importance of training employees in digitalization.

Reference [14] explores how Italian SMEs can overcome resistance to digitalization. The study finds that open innovation, training, networking, and co-creation initiatives play a crucial role in enhancing SMEs’ digital literacy and help them strategically adopt new digital technological solutions.

Reference [42] investigates factors influencing digital transformation in manufacturing. Larger firms adopt advanced technologies due to greater resources, while smaller firms benefit by focusing on high-value areas. High product innovation levels drive digital transformation, as innovative products require advanced manufacturing processes. Continuous production benefits from automation and analytics, while batch production focuses on flexible systems and digital supply chain management. Tailoring digital transformation strategies to specific firm characteristics and processes is crucial.

Reference [43] investigates the barriers and challenges that IT security poses to the digital transformation of SMEs, particularly in the manufacturing sector. This study highlights that SMEs often lack the necessary resources and expertise to effectively address these security challenges.

Reference [44] examines the factors influencing data analytics adoption in SMEs. The study identifies drivers such as improved decision-making, increased operational efficiency, and competitive advantage. Barriers include limited financial resources, a lack of skilled data professionals, and data privacy and security concerns.

Reference [45] analyzes the impact of various technological, organizational, and environmental prerequisites for the successful adoption of Artificial Intelligence (AI) technologies in manufacturing. The authors conclude that organizational factors, such as digital skills, company size, and R&D intensity, have the largest impact on the adoption of AI in manufacturing.

Reference [46] investigates how Canadian SMEs experience AI-driven digital transformation. The process unfolds in a gradual, path-dependent, and sequential manner, with leadership playing a recurring and omnipresent role.

Reference [47] explores the implementation of AI solutions tailored to the specific needs of SMEs in southwestern Finland. The study emphasizes the importance of early business and data understanding for the successful implementation of AI solutions that enhance business processes and benefits.

A branch of literature explores how SMEs built resilience through digital transformation during the COVID-19 pandemic [[48], [49]].

Finally, reference [50] indicates that the current literature lacks a fully developed roadmap for sustainable digital transformation in SMEs.

Based on the above findings, SMEs are expected to face challenges in digital transformation, with variations in the types of digital technologies they integrate. A positive correlation is anticipated between adoption or integration and factors such as size, digital infrastructure, and digitally skilled labor. However, regarding the latter, questions remain about digital skills, leadership and resistance. Additionally, given the lack of resources in SMEs, it is anticipated that value chain governance types that provide more resources (and less freedom) to SMEs, such as ‘Captive value chains’ and ‘Hierarchy’, will most effectively enable the adoption of Industry 4.0. This research tests these and other results related to the challenges faced by SMEs in the Portuguese metalworking industry in adopting Industry 4.0, using the AF method and regression analysis.

### 2.3 The European commission's digital transformation scoreboard

The EC's DTS framework is part of its Digital Transformation Monitor [51,52]. The DTS consists of three composite indicators that are made up of one or more categories or dimensions, and several partial indicators per dimension [51,52]. The outputs' composite indicators are the *Digital Technology Integration Index* (DTII) and the *ICT Start-up Evolution Index*, each of which only has one category or dimension [51,52]. The former measures the degree of digital technology integration in companies, other than start-ups, and considers eight partial indicators [52]. The latter measures the presence of digital start-up companies and considers four partial indicators [52].

**Fig. 1:**
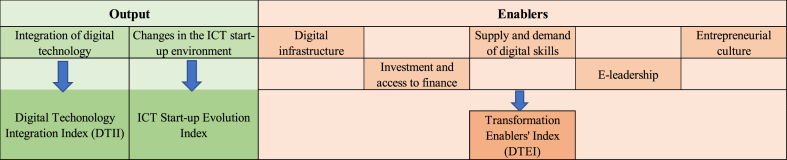
European Commission's DTS framework.

Source: Adapted from EC (2018)

The enablers' composite indicator is the *Digital Transformation Enablers' Index* (DTEI) [51,52]. It considers five dimensions and a total of 19 partial indicators [52]. The dimensions are: (i) ‘Digital infrastructure’, with the objective of grasping if it exists; (ii) ‘Investment and access to finance’, which aims to verify if there is any investment in activities linked to digitalization and how they are financed; (iii) ‘Supply and demand of digital skills’, which assesses the availability of digital capabilities among the population; (iv) ‘E-leadership’, which aims to understand the extent to which there is education and training that facilitates the creation of digital skills; (v) and ‘Entrepreneurial culture’, which aims to measure whether the national environment is business friendly and the level of business culture [51,52]. Fig. 1 provides an overview of the DTS framework.

Data for the calculation of partial indicators are collected in various databases and refer to all companies under analysis in each Member State (MS) [51,52]. The score of each MS in each partial indicator varies between 0 and 100 [51,52]. The outputs' composite indicators (DTII and ICT Start-up Evolution Index) only consider one dimension [51,52]. The enablers’ overall composite indicator (DTEI) considers five dimensions. For output and enablers, within each dimension, all partial indicators have the same weight [51,52].

### 2.4 The AF method

The AF method is a counting dual cut-off method developed to measure multidimensional poverty [[53], [54], [55], [56]]. However, it can be used for other purposes [e.g., 56, 63]. In this paper, it has been used to characterize the digital technology integration profiles of companies operating in the Portuguese metalworking industry (a sector dominated by SMEs), identifying the digital technologies that companies most lack, and computing partial and composite indicators, individual and average group, as well as group measures of digital technology integration, for benchmarking companies and groups of companies operating in the sector. The digital profiles of individual companies and the regression analysis also enable some results identified in the empirical literature on SMEs to be tested.

The AF method offers several advantages over alternative methods, such as Data Envelopment Analysis (DEA):

- Detailed individual profiles - The AF method allows for the generation of detailed individual profiles. Unlike some other approaches, it provides a comprehensive view of each individual under consideration.
- Sample size flexibility - Unlike DEA, which often requires large samples, the AF method is more flexible in terms of sample size. It can be applied effectively even with smaller datasets.
- Qualitative and quantitative information integration - The AF method can integrate both qualitative and quantitative data. This integration enhances the overall assessment process.
- Handling missing values - The AF method accommodates missing values more effectively. It ensures robustness in cases where data gaps exist.

Furthermore, in contrast to the DEA's subjective composite indicators (such as the ‘Benefit-Of-the-Doubt’ approach proposed in Ref. [57]), the AF method produces objective individual overall composite indicators. In this study, these indicators represent the proportion of digital technologies integrated by SMEs, with equal weights assigned to all partial indicators in the assessment.^4^

### 2.5 Research gaps addressed

This study contributes to the field by addressing the following research gaps: i) identifying the technologies integrated by SMEs in the Portuguese metalworking industry; ii) investigating factors driving this integration, including the identification of value chain governance types; and iii) utilizing and validating an existing research tool—the AF method—for analyzing Industry 4.0 integration by SMEs. This involves developing individual and group average partial and composite indicators, as well as group measures for benchmarking not only individual SMEs but also groups of SMEs.

## 3 Methodology

### 3.1 Implementation of the AF method

Regarding the integration of digital technology (output), the following approach was adopted to implement the AF method:

(i) only one dimension was considered for the output, in line with the EC's DTS, with a focus on 14 partial indicators;
(ii) the 14 partial indicators considered were dichotomized by applying critical cut-off values. Original scales of partial indicators were converted to 1 when the partial indicator is above the critical cut-off level (indicating good performance), and 0 otherwise (indicating poor performance). The cut-off levels correspond to the average of the original scale observations for each partial indicator;
(iii) missing values were filled with the value 0.5. This ensures that missing values align with the second set of critical cut-off values (in (v)) without affecting performance assessment decisions (in (vi)). In addition, the value 0.5 serves as the best estimate when no information is available;
(iv) equal weights were assigned to the partial indicators. This approach allows the individual overall composite indicator of digital technology integration to indicate the proportion of partial indicators where the company demonstrates good performance - an objective measure;
(v) the overall composite score for each company was computed, and the score obtained compared with the second set of critical cut-off values (set at 0.5) to determine satisfactory or unsatisfactory individual overall performance;
(vi) the average performance of a group of companies was calculated based on individual scores. These average scores for each partial and overall composite indicator were contrasted with the critical cut-off value of 0.5. An average score strictly above 0.5 indicates satisfactory performance for a specific partial indicator or the overall composite indicator; and
(vii) the following group measures were computed:
- H (Proportion): Identifying the proportion of companies with an overall composite score greater than the critical cut-off value 0.5;
- A (Intensity): Determining the average overall performance of the above group of companies;
- Group Digital Technology Integration Indicator (GDTII): Determining the group performance. The GDTII score results from multiplying H by A. A GDTII score strictly greater than 0.5 signifies satisfactory group performance.

It is also worth noting that a similar procedure was followed for each of the two enablers of the digital technology integration considered.

### 3.2 Development of the questionnaire

#### 3.2.1 Types of indicators considered

The questionnaire was developed by considering the types of indicators outlined in the EC's DTS, which serves as the primary reference for comparison, and by drawing insights from the literature surveyed. Additionally, the questionnaire incorporates the experience with SMEs at CATIM.^5^

#### 3.2.2 Sections of the questionnaire

The questionnaire had three sections. The first covered a few traits of the companies surveyed. The second characterized output and enabler dimensions of the digital transformation of the companies. The third was concerned with the types of value chain governance. The first and the third sections allowed a general characterization of the companies studied to be formed. The second section focused on the digital transformation of the companies.

Following the AF method, most questions in the questionnaire had closed Yes/No answers. The use of binary responses not only generated partial indicators that were easy to interpret, but also facilitated easier and faster responses from the companies, increasing the potential number of respondent companies, namely SMEs.

#### 3.2.3 General characteristics surveyed in the companies

In the first section, the data collected to characterize the companies surveyed comprised the following: CAE,^6^ company's age, dimension (number of workers and turnover), the turnover share of exports, number of customers, and location.

In the third section of the questionnaire, concerning the value chain governance, reference [58] typology was adopted and four questions with Yes/No answers were included to understand the governance of each company's value chain:

1. Does the company produce for a company that owns it?
2. Do the products sold by the company vary according to customers, i.e., are they customized and/or follow specifications given by customers?
3. Are the machines used in the production process specific, i.e., vary according to the specifications of the products given by the customers?
4. Does the company decide how the product is produced without any instructions from the customers?

Table 1 illustrates the links between the responses given by the companies to these questions and the value chain governance type.

**Table 1:** ‘Value chain governance’ identification.

‘Value chain governance’	Questions and answers			
	1	2	3	4
‘Markets’	No	No		
‘Modular value chains’	No	Yes	No	
‘Relational value chains’	No	Yes	Yes	Yes
‘Captive value chains’	No	Yes	Yes	No
‘Hierarchy’	Yes			

#### 3.2.4 Output and enabler dimensions and indicators of digital transformation

The data collected in the second section of the questionnaire focused on the digital transformation of companies. Concerning the development of the individual composite indicators, the AF method and the recommendations of reference [59] were considered.

As outputs of digital transformation, the DTII index of the EC's DTS [51,52] was adapted and extended to make it suitable for the company level and to be able to apply the AF method [[53], [54], [55], [56],^16^,^60^]. By applying the AF method, the overall composite indicator, referred to as the Digital Technology Integration Index (DTII), was obtained. This includes individual and group averages. Additionally, the group measure, known as the Group Digital Technology Integration Index (GDTII), was also obtained.

Concerning the integration of digital technology, 14 questions and/or partial indicators were considered:

1. Does the company use Enterprise Resource Planning (ERP) software or equivalent to share information across functional areas?

ERP is the integrated management of main business processes, mediated by software and technology [51,12]. The question aims to understand if there is the circulation of information within the organization mediated by software supporting decision-making processes.

2. Does the company use Radio-Frequency Identification (RFID) technology for product identification/monitoring? Alternatively, does the company use barcodes on products during production?

To establish a communication network among the various components of a factory, the first step is the integration and recognition of these same components in the network. The integration of location/identification devices into objects allows systems to identify objects. The location allows the monitoring and orientation of the products along the production line [51]. Barcodes are an alternative technology to RFID. They are especially important in the metalworking industry, as the metal may interfere with radio frequency.

3. Does the company use sensors to monitor products, for instance to measure temperature?

Sensors are a more advanced technology than RFID or barcodes because they allow information to be collected about an object and its environment at any given time, beyond location/identification [51].

4. Does the company use sensors to collect information on the machines employed in the production process?
Sensors are also important at the machine level, as they provide data that can be used to monitor the machines more effectively. For instance, vibration sensors can determine if a machine's rhythm is the desired one or if there is a problem. These sensors can also improve the efficiency of the production process by identifying the need to check machines before they develop a problem, making it possible to schedule machine maintenance in an optimal way [51].
5. Does the company use Customer Relationship Management (CRM) software to characterize customer profiles and better tailor and market its products?

This question is designed to determine if different customer information is used to improve products [51].

6. Does the company use any social networks?

Social networks are important for companies to be recognized and to analyze consumer preferences. This technology is more critical for companies that deal directly with final/end consumers [51].

7. Does the company use Electronic Data Interchange (EDI) type technology to send invoices to customers for automatic processing?

This question verifies the use of EDI-type technology. This technology allows data to be transferred from one computer to another, without human intervention, for automatic processing. It is mostly used in the manufacturing sector. It is also useful for instance to send production documents, easing the process of information exchange within value chains, and increasing the integration of technologies [51,52].

8. Does the company use cloud services to store information for the use of software as a service or for other purposes?

Cloud computing technology allows users to have access to a shared network of computing resources. By contracting cloud services, users do not have to invest in infrastructure and can take advantage of the technologies that best suit their business. Service providers promote standardization within their services, which is essential to ease the collaboration between different companies, a requirement for digitalization [52,61].

9. Does the company sell its products online?

Online sales are a stage of digitalization. Business-to-consumer (B2C) companies use online platforms as digital solutions to increase customer reach. The digitalization of communication with customers also allows the information generated to be shared with other activities of the value chain [51].

10. Does the company use Manufacturing Execution System (MES) software or equivalent?

MES or equivalent are computer programs that control, monitor, and report the state of production, working together with sensor and identification technologies. They allow the different activities of the production line to be integrated and automated while controlling production through the information that is incorporated and collected along the production line. Real-time production status documentation serves to assist decision-making in the production process by planning and controlling it effectively [62].

11. Does technology enhance the flexibility of the production line?

To explore the potential that digital integration offers, factories must have production lines that operate by modules and that allow the sequence of production to be changed according to needs [62].

12. Does the company share software or information systems, such as ERP, CRM, and MES or equivalent with customers or suppliers?

Extending the digitalization network beyond the company, to customers or suppliers, is important for analyzing the data of the entire value chain so that the interpretation of the data network is as complete as possible and incorporates other dimensions that affect the product and are not captured by the company [62].

13. Does the company analyze the information gathered from machine sensors to predict machine maintenance needs?

Digitalization is guided by information. Analysis of data from smart machine sensors can detect anomalies in the performance of machines before they fail, thus enabling fault prevention and increasing plant efficiency [62].

14. Does the company use data analysis to improve energy efficiency?

The flow of real-time data allows plants to be aware of current demand and to plan and manage machine usage to improve energy efficiency measures [62].

The above set of 14 questions or partial indicators enabled the development of a company profile in relation to their integration of digital technology.

**Fig. 2:**
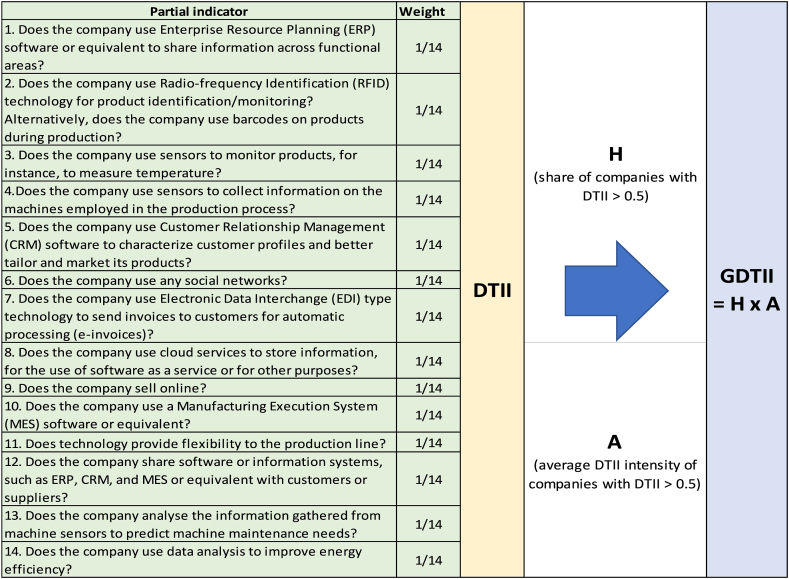
Digital technology integration partial indicators, DTII, and GDTII.

Source: Own work

Following the AF method [[53], [54], [55], [56],^16^,^60^], a value of 1 was assigned to a Yes answer, a value of 0 to a No answer. When no response was provided, a value of 0.5 was assigned. The average of the set of partial indicators indicated the degree of digital technology integration in the company.^7^ This composite indicator was referred to as the DTII. It was assumed that a value greater than 0.5 meant that the company had significantly integrated digital technology and that a value less than or equal to 0.5 meant the opposite.^8^ By following the AF method, it is possible to compute a measure of digital technology integration, for the sample of companies or subgroups of companies, as the product of the incidence H (the share of companies with DTII >0.5) and the average intensity A (the average DTII of companies with DTII >0.5). This measure was labelled the GDTII. Fig. 2 illustrates the digital technology integration partial indicators, the DTII, and the GDTII.

A procedure like the one illustrated in Fig. 2 was applied to each enabler dimension of digital transformation. Only two of the five dimensions of the EC DTS – digital infrastructure, and supply and demand of digital skills – were considered in the questionnaire as enabler dimensions of digital transformation. The enabler dimension of digital infrastructure aims to verify if companies have the key digital infrastructure to achieve the integration of digital technology. It is composed of four questions or partial indicators. The first two verify the existence of internet structures and the last two the existence of information management software:

1. Does the company have an internet connection with a speed of more than 30 MB?

The internet enables connectivity that allows the interaction of people and objects. Key technologies, such as cloud computing, IoT, social networking, mobile technologies, big data, and data analysis depend on the internet to some extent, as digitalization is mostly driven by data exchange. The speed of downloads and uploads is an essential prerequisite for its operation [51].

2. Does the company have a wireless internet network?

For successful digital transformation to take place, it is necessary for objects that are constantly moving to have an internet connection, which a wired network does not allow.

3. Does the company use any ERP-type software or equivalent?

An ERP-type software or equivalent is an important source of information [51]. It can add great value when a decision-making process needs to incorporate information about a company [63]. It contributes to digitalization by aggregating data on a company that can be useful for later incorporation into any activities of the value chain. In its most basic nature, it allows the digital organization of a company's information. In more advanced stages, it permits information from different sources to be compared, resulting in relevant interpretations for the companies and the application of the context-aware principle to the information, a principle that is very important for digital transformation [12].

4. Does the company use any CRM-type software or equivalent?

One of the main drivers of digitalization is the increasing importance of responding to the changing needs of customers. CRM-type software or equivalent allows the sharing of customer information within the organization. Customer needs are constantly being detected, which enables the organization to be flexible and to deal effectively with those needs [64].

The above-mentioned set of four questions or partial indicators enabled a company profile on digital infrastructure to be developed. Following the AF method [[53], [54], [55], [56],^16^,^60^] as previously carried out, the composite indicator *Digital Infrastructure Index* (DII) and the group measure *Group Digital Infrastructure Index* (GDII) were computed.

The supply and demand of the digital skills enabler dimension assess the skills of the labor force and/or the use by the labor force of information and communication technologies (ICTs) in the organization's operations. The four questions or partial indicators used were:

1. Do more than half of the employees have ICT skills?

It is understood that workers who have ICT skills are those that can work with ICT programs within the company. This might, for example, mean the ability to work with Excel, ERP or CRM software [51].

2. Does the company provide workers with ICT training?

As the supply of skilled labor is low and labor laws make it difficult to dismiss workers, training becomes especially important for adapting human resources to digitalization [51,64].

3. Does the company have difficulty recruiting or subcontracting human resources with ICT skills?

This question aims to understand if companies can access human resources with ICT skills through hiring or outsourcing [51]. Outsourcing these skills can be important for SMEs to access skills that they cannot internalize.

4. Do employees use portable devices provided by the company to communicate with each other and/or with the machines?

The interaction between people and machines is a central theme of digital transformation. Portable devices allow people to communicate, from anywhere, with each other and with machines, and they facilitate the implementation of agility, which is important for promoting flexible production and machine autonomy [51,12].

The above-mentioned set of four questions or partial indicators enabled a company profile on the supply and demand of digital skills to be developed. Following the AF method [[53], [54], [55], [56],^16^,^60^] as previously carried out, the composite indicator *Supply and Demand of Digital Skills Index* (SDDSI) and the group measure *Group Supply and Demand of Digital Skills Index* (GSDDSI) were computed.

### 3.3 Application and administration of the questionnaire

The population surveyed was a set of companies belonging to the Portuguese metalworking industry that were CATIM associates or customers, resulting in a universe of 239 companies.

The application of the online questionnaire involved three phases: (i) pre-pilot, (ii) pilot; and (iii) a final phase. The pre-pilot phase consisted of conducting the questionnaire in person with three companies. The objective of this first phase was to obtain feedback from these companies about the adequacy of the questions. The pilot phase involved sending the questionnaire to 100 randomly chosen companies other than the first three. This phase allowed final adjustments to the questions to be made. The final phase consisted of sending the questionnaire to the remaining 136 companies within the universe of 239 companies. In both the pilot and the final phases, after the first request for a response was made, two more reminders were sent to companies that had not yet responded. New response reminders were sent at intervals of 15 days.

For the pre-pilot phase, three SMEs were chosen based on convenience, and there was no need to extend the pre-pilot to more SMEs. As for the pilot, the questionnaire was sent to 100 companies to obtain a sufficiently large sample of respondent companies for the final adjustments to the questionnaire. The final phase sample corresponded to the remaining companies in the surveyed universe.

The questionnaire was administered between December 2018 and January 2019. Participation was voluntary, and anonymity was guaranteed for participating companies with respect to the reported information.

### 3.4 The OLS regression and the benchmarking exercise

The objective of the OLS regression was to evaluate the impact of some factors on the integration of digital technology by companies and identify subgroups of companies in the sample with different levels of digital technology integration to compare them using the group measures developed with the AF method. The regression equation used was the following:

(1) y=Xβ+ε

where y=DTII and X corresponds to the following regressors: *DII*, *SDDSI*, CAE dummies, company size based on the number of workers, company size based on turnover, the turnover share of exports, value chain governance dummies, and NUTS 3 location dummies.^9^ The latter dummies captured the effects of variables that were not observable but could be correlated with the regressors.

The dependent variable, *DTII*, is influenced by the explanatory variables or regressors. When the estimated coefficient of an explanatory variable is statistically significant, the sign of the coefficient indicates the direction in which the explanatory variable affects *DTII*. For instance, a positive coefficient suggests that an increase in the explanatory variable leads to an increase in *DTII*, and vice versa.

Additionally, this regression exercise enables the identification of distinct subsamples of companies within the sample based on their different *DTII* scores. These subsamples can then be compared or benchmarked using the group measures derived from the AF method.

## 4 Results and discussion

### 4.1 Sample representativeness

**Fig. 3:**
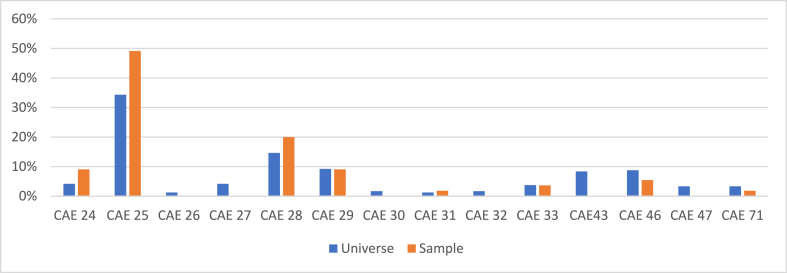
Distribution of the universe of companies surveyed and sample by CAE.

Source: Own work

Of the 239 companies surveyed, a total of 55 companies responded, which means a response rate of 23.0 %.^10^ To assess the representativeness of the sample of the overall population of companies surveyed, the Portuguese Classification of Economic Activities – Revision 3 (CAE-Rev.3) [65] was used, which provides the subsectors of the Portuguese metalworking industry. Fig. 3 illustrates the CAE distribution for both the entire population of surveyed companies and the sample.

The overall population of companies surveyed was distributed among 14 CAEs within the sector, while the sample of respondents was distributed across 8 CAEs. The CAEs in the overall population that were not represented in the sample had minimal impact. In addition, the most frequent CAEs in the sample – CAE 25 (‘manufacture of metal products, except for machinery and equipment’), CAE 28 (‘manufacture of machinery and equipment’), and CAE 29 (‘manufacture of motor vehicles, trailers, semi-trailers, and components for motor vehicles’) – were the most frequent CAEs in the overall population. The conclusions drawn indicated that the sample was reasonably representative of the overall population of companies surveyed.

### 4.2 General characteristics of sample companies

The average age of the companies in the sample was 35 years old. Overall, this was a group of companies that had been established for a long time. The age of the companies ranged from 6 to 97 years old. Of the 55 sample companies, 48 had fewer than 250 workers (87 %) and 50 had a turnover of less than €50 million (91 %). Therefore, most of the sample companies were SMEs.^11^ Only 45 companies responded to the question on the turnover share of exports. Almost half of the respondents (47 %) declared a share greater than 50 %. There were 49 responses to the question on the number of customers. The average number of customers of these companies was 600, but it ranged between 6 and 10,000.

It was possible to identify value chain governance in 49 sample companies. None of the companies responding indicated ‘Markets’. The most frequent types of value chain governance identified were the ‘Modular value chains’ (43 %), followed by the ‘Captive value chains’ (23 %), the ‘Hierarchy value chains’ (18 %), and the ‘Relational value chains’ (16 %). Most sample companies were from NUTS 3 AMP (Porto Metropolitan Area, 56 %), the territory where CATIM's headquarters are located, and from NUTS 3 Cávado (14 %), a neighboring territory.

### 4.3 Outputs and enablers of digital transformation

Table 2, Table 3 show the output and the enabler dimensions of digital transformation that were considered, respectively, indicating the group average scores of individual partial and composite indicators, and the scores of the group measures for the sample total (n = 55), the sample of SMEs (n = 50), and the sample of large companies (n = 5).^12^

**Table 2:** Outputs of digital transformation.

Outputs	Indicator	Sample (n = 55)	SMEs (n = 50)	LCs (n = 5)
Integration of digital technology	1. Does the company use Enterprise Resource Planning (ERP) software or equivalent to share information across functional areas?	0.745	0.730	0.900
	2. Does the company use Radio-Frequency Identification (RFID) technology for product identification/monitoring? Alternatively, does the company use barcodes on products during production?	0.582	0.540	1.000
	3. Does the company use sensors to monitor products, for instance to measure temperature?	0.327	0.260	1.000
	4. Does the company use sensors to collect information on the machines employed in the production process?	0.355	0.310	0.800
	5. Does the company use Customer Relationship Management (CRM) software to characterize customer profiles and better tailor and market its products?	0.355	0.340	0.500
	6. Does the company use any social networks?	0.709	0.680	1.000
	7. Does the company use Electronic Data Interchange (EDI) type technology to send invoices to customers for automatic processing (e-invoices)?	0.382	0.320	1.000
	8. Does the company use cloud services to store information for the use of software as a service or for other purposes?	0.573	0.530	1.000
	9. Does the company sell its products online?	0.091	0.100	0.000
	10. Does the company use a Manufacturing Execution System (MES) software or equivalent?	0.300	0.230	1.000
	11. Does technology provide flexibility to the production line?	0.545	0.510	0.900
	12. Does the company share software or information systems, such as ERP, CRM, and MES or equivalent with customers or suppliers?	0.191	0.140	0.700
	13. Does the company analyze the information gathered from machine sensors to predict machine maintenance needs?	0.309	0.280	0.600
	14. Does the company use data analysis to improve energy efficiency?	0.445	0.390	1.000
	DTII	0.422	0.383	0.814
	H	0.309	0.240	1.000
	A	0.702	0.656	0.814
	GDTII = H × A	0.217	0.157	0.814

**Table 3:** Enablers of digital transformation.

Enablers	Indicator	Sample (n = 55)	SMEs (n = 50)	LCs (n = 5)
Digital infrastructure	1. Does the company have an internet connection with a speed of more than 30 MB?	0.900	0.890	1.000
	2. Does the company have a wireless internet network?	0.982	0.980	1.000
	3. Does the company use any ERP-type software or equivalent?	0.791	0.780	0.900
	4. Does the company use any CRM-type software or equivalent?	0.627	0.610	0.800
	DII	0.825	0.815	0.925
	H	0.800	0.780	1.000
	A	0.912	0.910	0.925
	GDII = H × A	0.730	0.710	0.925
Supply and demand of digital skills	1. Do more than half of the employees have ICT skills?	0.582	0.580	0.600
	2. Does the company provide workers with ICT training?	0.636	0.620	0.800
	3. Does the company have difficulty recruiting or subcontracting human resources with ICT skills?	0.664	0.660	0.700
	4. Do employees use portable devices provided by the company to communicate with each other and/or with the machines?	0.664	0.630	1.000
	SDDSI	0.636	0.623	0.775
	H	0.545	0.540	0.600
	A	0.867	0.852	1.000
	GSDDSI = H × A	0.473	0.460	0.600

Concerning digital technology integration, the sample of SMEs (n = 50) was lagging relative to the sample of large companies (n = 5), as expected. Moreover, the average results of the sample total (n = 55) mainly reflect the average results of the sample SMEs (n = 50).

Concerning the integration of digital technology, the sample total had on average, a DTII = 0.422, which is below 0.5. By looking at the average scores of the partial indicators, it was possible to identify the main strengths and weaknesses. Most companies used a social network (0.709), they also used cloud services to store information for using the software as a service or for other purposes (0.573), and they had a flexible production line through technology (0.545). These are the strengths. The other partial indicators show weaknesses (average scores below 0.5). For example, most companies do not sell online (0.091) and do not share information systems, such as ERP, CRM, and MES or equivalent, with customers or suppliers (0.191). Moreover, the share of companies with DTII >0.5 is less than a third, with H = 0.309, A = 0.702, and GDTII = 0.217. It was concluded that, in general, the companies surveyed in the Portuguese metalworking industry, namely SMEs, had not yet fully completed the integration of digital technology.

Regarding the digital infrastructure enabler dimension, sample companies had on average, a DII = 0.825, which was well above 0.5. By looking at the average scores of the partial indicators, the reader can identify the main strengths and weaknesses. Moreover, the share of companies with DII >0.5 is four-fifths, with H = 0.800, A = 0.912, and GDII = 0.730. It was concluded that most of the metalworking industry companies surveyed, appear to have the basic digital infrastructure to enable the integration of digital technology.

Concerning the supply and demand of digital skills enabler dimension, sample companies had, on average, an SDDSI = 0.636, which was above 0.5. By looking at the average scores of the partial indicators, the reader can identify the main strengths and weaknesses. Moreover, the share of companies with SDDSI >0.5 was greater than half, with H = 0.545, A = 0.867, and GSDDSI = 0.473, leading to the conclusion that the workforce of most of these companies had digital skills that enabled the integration of digital technology. Nonetheless, companies needed to make more progress on this front than concerning digital infrastructure.

In summary, the results show that the integration of digital technology in the companies surveyed was still weak, especially in SMEs. They also show that most of the companies had the digital infrastructure (digital physical capital) that would enable this integration, but that they needed to improve the supply and demand of digital skills (digital human capital). These results align with the findings of references [41,14,30,36], which emphasize the necessity of developing human capital and capabilities to drive successful digital transformation. Reference [14] specifically identifies training, networking, and co-creation initiatives as instrumental in bolstering SMEs' digital literacy. These initiatives facilitate SMEs' strategic adoption of new technological solutions. The findings contribute to the ongoing discourse on SME digital transformation and inform policy development by highlighting both facilitators and obstacles. References [41,30] emphasize the significance of training employees in digitalization to attain elevated performance levels.

The above conclusions indicate some managerial implications. It seems evident that managers need to invest in the provision and allocation of adequate and affordable digital infrastructure and platforms alongside the identification of the skills required for the implementation of Industry 4.0 and need to reach a compromise vis-à-vis the negotiations of the investment in technology and skills (adaptation and/or recruitment), surrounding organizational and cultural barriers. Consistent with these findings, references [39,30,46] highlight the pivotal role of leadership in digital transformation. Moreover, it is important to note that investment in skills recruitment and adaptation is never automatic or immediate. It requires medium to long-term planning. SME managers need to develop and implement a coherent and comprehensive digital strategy and agenda for designing and enforcing appropriate and effective digital regulation and standards, training, and recruitment.

The existence of organizational and cultural barriers, namely regarding the use of digital human capital, the integration of the industry SMEs in certain types of relationships within the value chains in which they participate, and the cooperation between firms in the industry are important avenues for future research. Given the supply chain disruptions because of the COVID-19 pandemic and the Russian-Ukraine conflict, managers were bound to be creative in finding new suppliers and adapting their products and production processes according to the availability of materials. This context might have influenced the pace of integration of digital technology in many of the companies surveyed. The online questionnaire was launched before the COVID-19 pandemic. It would be interesting to evaluate the impact of the pandemic and the war on the industry's integration of digital technology. And for that, the proposed AF method could be used again in the companies surveyed and the results benchmarked with those obtained in this research.

### 4.4 The OLS regression and the benchmarking exercise

Table 4 shows the OLS regression results. NUTS 3 territorial dummies are not stated in the table.^13^

**Table 4:** Factors affecting the integration of digital technology.

Variable	Coefficient
Constant	−0.113
DII	0.450∗∗
SDDSI	0.120
Relational value chains	−0.020
Captive value chains	0.164∗∗
Hierarchy	0.018
CAE 28	−0.146∗∗
CAE 29	0.026
CAE 31	−0.223∗∗
CAE 33	0.026
CAE 46	−0.235∗∗
CAE 71	−0.256∗∗
Age of the company	−0.002
Number of workers	−0.002
Turnover	0.051
Turnover share of exports	0.091
Number of clients	−5.930e-06
Number of observations 55	
Adjusted R-squared 0.530	

As expected, the DII enabler dimension had a positive and significant relationship with the integration of digital technology (DTII). The coefficient in the SDDSI enabler dimension was positive, but not significant. Possible explanations for this last result are the organizational difficulties of companies in finding and using qualified digital workforce and/or cultural barriers, such as the lack of support from the SME top management, the resistance of employees to adopt Industry 4.0 and the lack of cooperation between companies in the sector.

While further study is necessary, the results are aligned with the existing literature. Reference [45] finds evidence that ‘organizational factors, such as digital skills, company size, and R&D intensity, have the greatest impact on the adoption of AI in manufacturing’. They affirm the ‘high relevance of digital skills in explaining the absorptive capacity to improve the use of new technologies.’ Additionally, reference [31] demonstrates the relationship between digital adoption, ecosystems, and the performance of SMEs. Furthermore, reference [33] also concludes that prioritizing research and skilled human capital alongside digitalization can lead to better SME innovation outcomes. The lack of significance of digital skills in explaining the absorptive capacity to enhance the use of new technologies in our results may be attributed to the need for greater and more effective investment in digital skills, as well as to organizational and cultural barriers that hinder their proper utilization.

Regarding value chain governance, only captive value chains were associated with greater integration of digital technology. The coefficient was positive and significant. Of the eleven companies in the sample that operated in this type of value chain, three were large companies and eight were SMEs. The results are in line with expectations.

Except for the ‘manufacture of machinery and equipment’ (CAE 28), with eleven observations (one large company and ten SMEs), the other significant CAEs had very few observations. The coefficient in CAE 28 was negative and significant. Companies in this sub-sector had less integration of digital technology.

Given the OLS regression results, three subgroups of companies have been benchmarked with the sample total, using the GDTII group measure developed following the AF method:

a) Companies with DII >0.5.
b) Companies with captive value chains.
c) Companies belonging to CAE 28.

Table 5 shows the results of the benchmarking exercise.

**Table 5:** Group digital technology integration index.

Companies (#)	H	A	GDTII
Sample (55)	0.309	0.702	0.217
Companies with DII >0.5 (44)	0.364	0.711	0.259
Companies with captive value chains (11)	0.636	0.691	0.439
Companies belonging to CAE 28 (11)	0.273	0.755	0.206

As expected, the two subgroups of companies – companies with DII >0.5 and companies with captive value chains – had a GDTII greater than the sample totals, while the third subgroup of companies – companies belonging to CAE 28 – had a GDTII smaller than the sample totals. In the first subgroup, a higher share of H and intensity of A explain the positive difference. In the second (third) subgroup, it is a higher (lower) share of H that justifies the positive (negative) discrepancy.

These differences appear to align with the conclusions of recent work in Ref. [17], which studies the adoption of industrial robots in two main segments of the automotive value chain and concluded that there is significant heterogeneity in technology adoption within the same sector. The authors argue that other context-specific factors of the local ecosystem may have an important influence on the adoption of industrial robots. More recently, reference [40] concludes that both market pressures and organizational readiness exert a substantial influence on the adoption of digital marketing strategies by SMEs. This conclusion corroborates our conclusions examining the determinants of digital transformation in SMEs.

The foregoing conclusions have managerial implications. It seems evident that managers need to invest in the provision and allocation of adequate and affordable digital infrastructure and platforms alongside the identification of the skills required for the implementation of Industry 4.0 and need to reach a compromise vis-à-vis the negotiations of the investment in technology and skills, surrounding organizational and cultural barriers. Furthermore, it is crucial to recognize that investing in skill recruitment and adaptation is not a spontaneous or short-term process. It necessitates medium to long-term planning. SME managers must develop and implement a cohesive digital strategy and agenda that encompasses the design and enforcement of suitable digital regulations, standards, training, and recruitment initiatives.

In terms of public policy, these results suggest the potential for a tax incentive scheme, based on labor taxes and associated with Industry 4.0 investment in equipment combined with investment in people reskilling, new skills recruitment, and investment in IT security [43]. Tax incentives could also be used to promote companies' cooperation in the industry. Moreover, governments, regulators, and agencies can create and improve the conditions and incentives for DTI in SMEs, through the development and implementation of a coherent and comprehensive digital strategy and agenda for SMEs, the provision and allocation of adequate and affordable digital infrastructure and platforms, and through the design and enforcement of appropriate and effective digital regulation and standards for SMEs. This suggestion is corroborated by the research in Ref. [38], which underscores the government's pivotal role in influencing technology adoption patterns.

## 5 Conclusion

### 5.1 Main findings, implications, and contributions

In this research, the state of digital technology integration in companies operating in the Portuguese metalworking industry, a sector dominated by SMEs, has been examined and assessed using the AF method. For this purpose, at the company level, the type of indicators established by the EC DTS has been adapted and extended, and an online questionnaire carried out.

Results show that the integration of digital technology has been weak (GDTII = 0.217, n = 55) and that SMEs have been lagging (GDTII = 0.157, n = 50) when it comes to outputs and enablers of digital transformation. Companies have the basic digital infrastructure to enable the integration of digital technology (GDII = 0.730), but they need to improve their supply and demand for digital skills (GSDDSI = 0.473).

By looking at the average scores of the partial indicators, it was possible to identify the main strengths and weaknesses. Most companies make use of a social network (0.709), they also use cloud services to store information for using the software as a service or for other purposes (0.573), and they have a flexible production line through technology (0.545). These are the strengths. The other partial indicators are weaknesses (average scores below 0.5). For example, most companies do not sell online (0.091) or share information systems, such as ERP, CRM, and MES or equivalent, with customers or suppliers (0.191). Moreover, the share of companies with a digital integration indicator (DTII) above 0.5, is less than a third.

Regarding the regression analysis, the DII enabler dimension affected positively and significantly the integration of digital technology in companies, as expected. The enabler dimension SDDSI shows a positive, but not significant relationship, which may denote the organizational difficulties and/or cultural barriers SMEs face concerning Industry 4.0. This means it is not enough for companies to have digital skills, they need to be able to use them. Nonetheless, the result is not inconsistent with more recent findings about the prerequisites for the adoption of AI technologies in manufacturing.

The research also contributes to the development of a new framework for identifying the value chain governance types proposed in Ref. [58]. Captive value chains were associated with greater integration of digital technology within industry companies. However, they also correlate with reduced autonomy for SME top management. Consequently, cultural barriers may deter SMEs from joining value chains characterized by this type of relationship.

From the results of the regression analysis, three subgroups of companies in the sample were identified and benchmarked against the sample totals: two with higher integration of digital technology (companies with a high DII and companies with a captive value chain), and one with a lower integration of digital technology (companies in the ‘manufacturing of machinery and equipment’ subsector, with an even greater predominance of SMEs). This observed heterogeneity is also consistent with the literature.

The research has significant managerial implications. SME managers must develop and implement a coherent and comprehensive digital strategy and agenda, focusing on designing and enforcing appropriate and effective digital regulations and standards, as well as on training and recruitment. Additionally, the research has public policy implications. The potential for tax schemes to help overcome organizational and cultural barriers to the digital transformation of the industry is evident.

Finally, the research also contributes by utilizing an existing research tool, the AF method, to analyze the adoption of Industry 4.0. As shown, the AF method has several advantages: (i) it does not require very large samples; (ii) it accommodates missing values more easily, enabling the production of objective composite indicators (namely the proportion of digital technologies integrated by companies); (iii) it is easily implemented by companies, namely SMEs; and (iv) it generates not only individual and average group detailed profiles and composite indicators, but also group measures, allowing companies and groups of companies to be more effectively benchmarked. The AF method is easy to understand and apply by policy makers, and the detailed information it produces can be useful for policy design.

### 5.2 Limitations and future research

There are several limitations to this research. Firstly, the set of partial indicators considered could be enhanced by incorporating recent developments in the integration of digital technology within companies.

Secondly, the universe of companies surveyed consists of clients and associates of CATIM. While the sample of respondent companies is representative of this universe, it may not necessarily reflect the entire metalworking industry population in Portugal. Extending the questionnaire to include all companies within the Portuguese metalworking industry would allow for a better understanding of the broader Portuguese context.

Thirdly, the survey was conducted before the COVID-19 pandemic. Given that the pandemic and the Russia-Ukraine war accelerated digital technology adoption in many companies, it would be interesting for future research to explore how the Portuguese metalworking industry has evolved in terms of digital technology integration.

Another emerging topic worth exploring in future research is the connection between digital transformation and the sustainability of SMEs in the Portuguese metalworking industry.

Lastly, benchmarking the Portuguese metalworking industry against other European Union countries using the AF method could provide valuable insights for future studies.

## CRediT authorship contribution statement

**Luís L. Moreira:** Writing – original draft, Validation, Project administration, Investigation, Formal analysis, Data curation, Conceptualization. **Sofia S. Pinto:** Writing – review & editing, Writing – original draft, Validation, Supervision, Investigation, Formal analysis, Conceptualization. **Leonardo Costa:** Writing – review & editing, Validation, Supervision, Project administration, Methodology, Investigation, Funding acquisition, Formal analysis, Conceptualization. **Nuno Araújo:** Writing – review & editing, Validation, Resources, Project administration, Formal analysis.

## Ethics declaration

The article's publication is approved by all authors. The work described has not been published previously. The article is not under consideration for publication elsewhere.

## Data availability statement

Data will be made available on request.

## Declaration of competing interest

The authors declare that they have no known competing financial interests or personal relationships that could have appeared to influence the work reported in this paper.

## Footnotes

3 Reference [58] classifies value chain governance into five types, depending on the complexity of the transactions, the ability to codify knowledge, and the skills of the suppliers: ‘Markets’, ‘Modular value chains’, ‘Relational value chains’, ‘Captive value chains’, and ‘Hierarchy’ [32, p. 83–84].

## Footnotes

1 Refer to Ref. [66] for the European Commission's definition of SMEs.

2 According to the European Commission [67], in 2022, SMEs in the EU-27 accounted for 99.8 percent of all enterprises, 64.4 percent of employment, and 51.8 percent of the value added in the non-financial business economy.

4 See Ref. [68] for a literature survey on composite indicators of company performance.

5 CATIM - Technological Center for the Metal Working Industry - is a technological centre that supports the Portuguese metalworking industry. See https://www.catim.pt/en.

6 CAE is the Portuguese Classification of Economic Activities [65].

7 Assuming equal weights for the partial indicators means that the DTII indicates the proportion of ‘digital technologies’ integrated by the company.

8 With 0.5 being the cut-off level for each company's DTII, assigning a value of 0.5 to missing partial indicator values does not affect the company's position regarding its integration of digital technology (indicated by the corresponding DTII), and it is the best guess in the absence of any information. However, a sensitivity analysis was conducted by assigning a value of 1 or 0 to the missing data points. The purpose was to verify whether the results changed significantly, and they did not.

9 The NUTS classification (Nomenclature of Territorial Units for Statistics) is a hierarchical system for dividing up the economic territory of the European Union [69]. NUTS 3 corresponds to small regions or subregions.

10 The response rate for the online questionnaire is acceptable. Additionally, given a sample size of 55 observations, the Central Limit Theorem applies. This means that it is possible to make inferences from the sample to the population of companies surveyed.

11 On SMEs' definition, see the Commission Recommendation of 6 May 2003 [66].

12 The missing values sensitivity analysis is available upon request.

13 The full results of the OLS regression in GRETL are available upon request.
