# Supplementary material for: Evaluating digital transformation in small and medium enterprises using the Alkire-Foster method
Source: Heliyon. 2025 Jan 10;11(2):e41838. doi: 10.1016/j.heliyon.2025.e41838 (PMC11783441; doi:10.1016/j.heliyon.2025.e41838)
Supplement: Multimedia component 1 [file mmc1.pdf]

centro de apoio tecnológico
à indústria metalomecânica

CATÓLICA PORTO BUSINESS SCHOOL

PORTO

Transformação digital no setor metalomecânico em Portugal

Questionário

Secção 1 – Caraterização geral da empresa

Identificação da empresa	
Nome	
Concelho	
Ano de constituição	

Responsável pelo preenchimento do questionário	
Nome	
Função	
Contacto (telefone/e-mail)	

Atividade da empresa em 2017	Valor			
Produtos principais (descrição)				
Volume de negócios (milhões de €, assinalar intervalo aplicável com x)	Menos de 2	2 a menos de 10	10 a menos de 50	50 ou mais
Percentagem das exportações no volume de negócios (%)				
Número de colaboradores (assinalar intervalo aplicável com x)	Menos de 10	10 a menos de 50	50 a menos de 250	250 ou mais
Número aproximado do total de clientes (nº)				
Percentagem de novos clientes no total (%)				

Secção 2 – Transformação digital da empresa

*** (No final do questionário encontra um conjunto de breves notas explicativas de suporte ao preenchimento desta Secção 2).**

Dimensão	Questão	Sim	Não	NR/NS
**1. Infraestrutura digital**	1.1 A empresa tem conexão à internet com velocidade superior a 30 MB/s?			
	1.2 A empresa tem rede de internet sem fios?			
	1.3 A empresa usa algum *software* do tipo ERP ou equivalente?[1]			
	1.4 A empresa usa algum *software* de apoio à gestão de clientes?[2]			
**2. Competências digitais da força laboral**	2.1 Mais de metade dos colaboradores tem competências em TIC (tecnologias de informação e comunicação)?[3]			
	2.2 A empresa proporciona aos trabalhadores formação em TIC?			
	2.3 A empresa tem dificuldade em recrutar ou subcontratar recursos humanos com competências em TIC?[4]			
	2.4 Os colaboradores usam aparelhos portáteis fornecidos pela empresa para comunicarem entre si e/ou com as máquinas?[5]			
**3. Integração da tecnologia digital**	3.1 A empresa usa um *software* do tipo ERP ou equivalente para partilhar informações entre áreas funcionais?[6]			
	3.2 A empresa usa código de barras nos produtos durante a produção?[7]			
	3.3 A empresa usa tecnologia do tipo RFDI/ identificação por radiofrequência para identificação/monitorização dos produtos?			
	3.4 A empresa usa sensores, por exemplo, de temperatura para a monitorização dos produtos?[8]			
	3.5 A empresa usa sensores para recolher informação sobre as máquinas utilizadas no processo produtivo?[9]			

	3.6 A empresa usa um *software* de apoio à gestão de clientes para caracterizar os perfis dos mesmos e para adequar e comercializar melhor os seus produtos?[10]			
	3.7 A empresa usa alguma rede social?			
	3.8 A empresa usa tecnologia do tipo EDI ou equivalente no envio aos clientes de faturas para processamento automático (e-faturas)?[11]			
	3.9 A empresa usa serviços de *cloud* para armazenamento de informação, para uso de *software as a service* ou para outros fins?[12]			
	3.10 A empresa faz vendas online?			
	3.11 A empresa usa algum *software* do tipo MES (*Manufacturing Execution Systems*) ou equivalente?[13]			
	3.12 A linha de produção é flexível com recurso à tecnologia?[14]			
	3.13 A empresa partilha *softwares*/sistemas de informação, como CRM, MES e/ou ERP ou equivalentes, com clientes ou fornecedores?[15]			
	3.14 A empresa analisa a informação recolhida dos sensores das máquinas/objetos para prever a necessidade de manutenção das máquinas?[16]			
	3.15 A empresa utiliza a análise de dados para melhorar a eficiência energética?[17]			

Secção 3 – Relação da empresa na cadeia de valor

Questão	Sim	Não	NR/NS
A empresa produz para uma empresa que é sua proprietária?			
Os produtos vendidos pela empresa variam consoante os clientes, isto é, são customizados e/ou seguem especificações dadas pelos clientes?			
As máquinas usadas no processo de produção são específicas, isto é, variam de acordo com as especificações dos produtos dadas pelos clientes?			
A maneira de produzir os produtos é decidida pela empresa, sem qualquer instrução dos clientes?			

A empresa tem muita rotatividade de clientes?			

FIM

[1]1.3 *Software* do tipo ERP - *Enterprise Resource Planning*. Um *software* deste tipo ou equivalente organiza e integra a informação das diferentes áreas funcionais da empresa, apoiando a gestão de informação e a tomada de decisão. Pretende-se saber se a empresa usa este tipo de ferramentas para apoio à gestão de informação e tomada de decisão.

[2] 1.4 *Software* do tipo CRM - *Customer Relationship Management*. Um *software* deste tipo ou equivalente regista informações sobre o perfil dos clientes como (ex: preocupações dos clientes, compras realizadas e outras informações). A ferramenta permite à empresa melhorar as relações com os clientes e apresentar propostas ou soluções de produção mais adequadas ao perfil dos mesmos.

[3] 2.1 Pretende-se saber se a maioria dos colaboradores da empresa possui competências em TIC que facilitem o desempenho das suas funções na empresa. Dependendo das funções, estas competências podem ser no uso do Excel, do Word, do CAD, do CNC ou de outros *softwares* do tipo CRM e/ou o do tipo ERP ou equivalentes.

[4]2.3 Pretende-se saber se a empresa tem dificuldade em ter acesso a recursos humanos com competências em TIC, quer através de recrutamento, quer através do recurso a *outsourcing*.

[5]2.4 Pretende-se saber se há funções ao nível da empresa que são facilitadas através da interação via aparelhos portáteis com outros colaboradores e/ou com as máquinas. As interações com as máquinas podem traduzir-se em acesso a interfaces que permitem a visualização de informação das máquinas ou, através do aparelho portátil, ordenar a execução de comandos, como ligar e desligar.

[6]3.1 Pretende-se saber se a empresa usa um *software* do tipo ERP ou equivalente para apoio à tomada de decisão e/ou no desempenho de tarefas em determinadas áreas funcionais com recurso a informações que foram recolhidas noutras áreas.

[7]3.2 Pretende-se saber se a empresa usa a tecnologia de código de barras como alternativa à rádio frequência, uma vez que o metal pode interferir com a rádio frequência.

[8]3.4 Pretende-se saber se a empresa usa sensores na monitorização dos produtos durante a produção, distribuição ou pós-venda. Estes sensores podem ser de temperatura, de proximidade, de movimento ou outros.

[9]3.5 Pretende-se saber se a empresa usa sensores para monitorizar as máquinas e recolher informação sobre as mesmas que possa mais tarde ser utilizada pela empresa.

[10]3.6 Pretende-se saber se a empresa usa a informação registada nos *softwares* do tipo CRM - *Customer Relationship Management* – ou equivalente para fazer novas propostas de produção aos clientes existentes ou a novos clientes.

[11]3.8 A tecnologia EDI - *Electronic Data Interchange* – ou equivalente permite a transferência de dados organizados de acordo com determinadas diretrizes de um computador para outro computador, sem intervenção humana, como é o caso das e-faturas.

[12]3.9 Pretende-se saber se a empresa usa a tecnologia *cloud*. Esta tecnologia permite armazenar dados ou usar serviços, como *softwares* ou *interfaces*, que são geridos por uma empresa especializada externa.

[13]3.11 Pretende-se saber se a empresa usa algum *software* do tipo MES - *Manufacturing Execution Systems* – ou equivalente. Estes são *softwares*/programas computorizados que monitorizam e relatam o estado da produção de um produto em tempo real, desde a ordem de produção até o produto estar finalizado.

[14]3.12 Pretende-se saber se a tecnologia da empresa permite alterar a sequência do processo de produção do produto de acordo com as especificações da encomenda.

[15]3.13 Pretende-se saber se há integração e difusão da informação entre as várias empresas envolvidas na produção do produto final, através da partilha dos sistemas de informação.

[16]3.14 Pretende-se saber se a empresa recorre à análise dos dados recolhidos das máquinas para planear e agendar a sua manutenção.

[17]3.15 Pretende-se saber se a empresa consegue melhorar a fatura energética com o recurso à análise de dados, de forma a melhorar o planeamento da produção.
