# Supplementary material for: Evaluating digital transformation in small and medium enterprises using the Alkire-Foster method
Source: Heliyon. 2025 Jan 10;11(2):e41838. doi: 10.1016/j.heliyon.2025.e41838 (PMC11783441; doi:10.1016/j.heliyon.2025.e41838)
Supplement: Multimedia component 2 [file mmc2.pdf]

centro de apoio tecnológico
à indústria metalomecânica

CATÓLICA
CATÓLICA PORTO BUSINESS SCHOOL
PORTO

Digital Transformation in the Mechanistically Sector in Portugal

Questionnaire

Section 1 – General characterization of the company

Identification of the company	
Name	
County	
Year of constitution	

Person responsible for completing the questionnaire	
Name	
Role in the company	
Contact (telephone/e-mail)	

Company activities in 2017	Value			
Main products produced (description)				
Turnover (in millions of euros, indicate applicable range with x)	Less than 2	2 to less than 10	10 to less than 50	50 or more
Percentagem das exportações no volume de negócios (%)				
Number of employees (indicate applicable range with x)	Less than 10	10 to less than 50	50 to less than 250	250 or more
Approximate total number of clients (#)				
Percentage of new clients in the total (%)				

Section 2 – Digital transformation of the company

*** (At the end of the questionnaire, you will find a set of brief explanatory notes to support the completion of this Section 2).**

Dimension	Question	Yes	No	NR / NK
**1. Digital infrastructure**	1.1 Does the company have an internet connection with a speed of more than 30MB?			
	1.2 Does the company have a wireless internet network?			
	1.3 Does the company use any ERP-type software or equivalent?[1]			
	1.4 Does the company use any CRM-type software or equivalent?[2]			
**2. Digital skills of the labor force**	2.1 Do more than half of the employees have ICT skills?[3]			
	2.2 Does the company provide workers with ICT training?			
	2.3 Does the company have difficulty recruiting or subcontracting human resources with ICT skills?[4]			
	2.4 Do employees use portable devices provided by the company to communicate with each other and/or with the machines?[5]			
**3. Integration of digital technology**	3.1 Does the company use Enterprise Resource Planning (ERP) software or equivalent to share information across functional areas?[6]			
	3.2 Does the company use barcodes on products during production?[7]			
	3.3 Does the company use Radio-Frequency Identification (RFID) technology for product identification/monitoring?			
	3.4 Does the company use sensors to monitor products, for instance, to measure temperature?[8]			
	3.5 Does the company use sensors to collect information on the machines employed in the production process?[9]			
	3.6 Does the company use Customer Relationship Management (CRM) software to characterize customer profiles and better tailor and market its products?[10]			

	3.7 Does the company use any social networks?			
	3.8 Does the company use Electronic Data Interchange (EDI) type technology to send invoices to customers for automatic processing?[11]			
	3.9 8.Does the company use cloud services to store information for the use of software as a service or for other purposes?[12]			
	3.10 Does the company sell its products online?			
	3.11 Does the company use a Manufacturing Execution System (MES) software or equivalent?[13]			
	3.12 Does technology provide flexibility to the production line?[14]			
	3.13 Does the company share software or information systems, such as ERP, CRM, and MES or equivalent with customers or suppliers?[15]			
	3.14 Does the company analyse the information gathered from machine sensors to predict machine maintenance needs?[16]			
	3.15 Does the company use data analysis to improve energy efficiency?[17]			

Section 3 – Value chain governance

Question	Yes	No	NR/NK
Does the company produce for a company that owns it?			
Do the products sold by the company vary according to customers, i.e., are they customized and/or follow specifications given by customers?			
Are the machines used in the production process specific, i.e., vary according to the specifications of the products given by the customers?			
Is the way the company produces the product decided by the company without any instructions from the customers?			
The company has a lot of customer churn?			

END

[1]1.3 ERP Software - Enterprise Resource Planning: Software of this type or equivalent organizes and integrates information from different functional areas of the company, supporting information management and decision-making. The goal is to determine whether the company uses such tools for information management and decision support.

[2] 1.4 CRM Software - Customer Relationship Management: Software of this type or equivalent records information about customer profiles (e.g., customer concerns, purchases made, and other details). The tool enables the company to improve customer relationships and propose production solutions tailored to their profiles.

[3] 2.1 Skills in Information and Communication Technology (ICT): We aim to understand whether the majority of company employees possess ICT skills that enhance their performance in various roles. Depending on their functions, these skills may include proficiency in Excel, Word, CAD, CNC, or other CRM and/or ERP software equivalents.

[4]2.3 Access to ICT Talent: We want to know if the company faces challenges in accessing human resources with ICT competencies, either through recruitment or outsourcing.

[5]2.4 Portable Device Interactions: Does the company use portable devices to facilitate interactions among employees or with machines? These interactions could involve accessing machine information interfaces or issuing commands (e.g., turning on or off) via portable devices.

[6]3.1 ERP Software Usage for Decision Support: Does the company use ERP software or equivalent tools to support decision-making and perform tasks in specific functional areas, leveraging information collected from other areas?

[7]3.2 Barcode Technology Usage: Is barcode technology used as an alternative to radio frequency, especially considering potential interference caused by metal?

[8]3.4 We want to know if the company uses sensors to monitor products during production, distribution, or after-sales. These sensors can be related to temperature, proximity, motion, or other factors.

[9]3.5 We want to know if the company use sensors to monitor machines and collect information about them for later use?

[10]3.6 We want to know if information recorded in CRM software (Customer Relationship Management) or equivalent used by the company to make new production proposals to existing or new clients?

[11]3.8 The technology of Electronic Data Interchange (EDI) or equivalent allows the organized transfer of data from one computer to another without human intervention, as seen in e-invoices.

[12]3.9 We want to know if the company utilizes cloud technology. This technology enables data storage or the use of services, such as software or interfaces, managed by an external specialized company.

[13]3.11 We want to know if the company uses Manufacturing Execution Systems (MES) software or equivalent. These computerized programs monitor and report real-time production status, from production orders to the final product.

[14]3.12 We want to know if the company's technology allows to adjust the production process sequence according to order specifications?

[15]3.13 We want to know if there is information integration and dissemination among the various companies involved in the production of the final product through shared information systems?

[16]3.14 We want to know if the the company analyzes data collected from machines to plan and schedule maintenance?

[17]3.15 We want to know if the company improves its energy bill through data analysis to enhance production planning.
